# ADHD and Academic Success in University Students: The Important Role of Impaired Attention

**DOI:** 10.1177/10870547211036758

**Published:** 2021-08-12

**Authors:** Colin Henning, Laura J. Summerfeldt, James D. A. Parker

**Affiliations:** 1 Trent University, Peterborough, ON, Canada

**Keywords:** ADHD, post-secondary success, academic success

## Abstract

**Objective:**

To improve on several methodological issues regarding current literature investigating the relationship between ADHD symptomatology and academic success in adults and examine the relative contributions of specific dimensions of ADHD symptomatology (i.e., inattention, hyperactivity, and impulsivity) to post-secondary academic success.

**Method:**

A large sample of 3,688 post-secondary students were examined using a longitudinal design. The Conners’ Adult ADHD Rating Scale (CAARS) was used to assess adult ADHD symptoms and academic success was assessed using students’ official academic records (e.g., final GPAs and degree completion status).

**Results:**

Students with greater inattention symptomatology at the start of their academic program showed consistently poorer long-term academic success (i.e., lower GPAs, higher dropout rates), regardless of gender.

**Conclusion:**

Inattention symptoms are the primary driver of the relationship between ADHD symptomatology and academic underachievement in adults. Post-secondary education institutions should target and prioritize educational programming for inattention symptoms of ADHD in at-risk post-secondary students.

ADHD is defined in the DSM-5 as a persistent pattern of inattention and/or hyperactivity-impulsivity that shows clear evidence of interference with social, academic, or occupational functioning in two or more settings (e.g., in the home, at school; American Psychiatric Association [APA], 2013). ADHD is primarily characterized by a set of core symptoms along two related dimensions: inattention (e.g., difficulty focusing on a task at hand) and hyperactivity-impulsivity (e.g., restlessness and inability to wait). ADHD symptomatology has been consistently associated with several adverse life outcomes, including academic underachievement, higher rates of unemployment and precarious employment, increased rates of substance use disorders, and reduced quality of life (Cherkasova et al., 2013; Erskine et al., 2016; Kuriyan et al., 2013).

## ADHD and Academic Achievement

Perhaps the most extensively studied of these adverse outcomes of ADHD is its impact on a person’s ability to succeed in educational settings (Kent et al., 2011; Lawrence et al., 2021). Indeed, in a study examining the academic achievement of children and adolescents, Lawrence et al. (2021) found that children with ADHD were, on average, a year behind their non-ADHD peers on standardized tests for reading and math by their third year of schooling, and adolescents with ADHD were, on average, 2.5 years behind their non-ADHD peers on standardized tests for reading and 3 years behind on standardized tests of math by their ninth year of schooling. Likewise, Kent et al. (2011) found that secondary school students with ADHD received lower overall grade point averages (GPAs) and lower GPAs in all academic domains compared to their non-ADHD peers, as well as being less likely to hand in completed assignments on time and more likely to fail courses throughout secondary school and drop-out prematurely.

Despite such consistent evidence regarding the academic difficulties of children and adolescents with an ADHD diagnosis, research regarding the academic achievement of adults in post-secondary education has been somewhat less consistent and robust. For instance, Gray et al. (2016) found no evidence of impairment in academic performance among adults with diagnosed ADHD on standardized tests of executive functioning and in GPAs. However, this study employed a cross-sectional design that limited its examination of relevant academic outcomes and relied on students’ self-reported GPAs which are subject to response biases. Similarly, research by Lewandowski et al. (2008) found that reported academic concerns were neither sensitive nor specific to ADHD diagnosis. Unfortunately, this study employed a convenience sample of students ranging in age from 18 to 49, thus the results are likely confounded by the reduction in ADHD symptomatology generally observed over the course of adulthood (Kim et al., 2015).

Prevatt and Young (2014), on the other hand, have reported evidence from several studies that college students with ADHD receive lower grades than their non-ADHD peers, and are more likely to withdraw from classes, have poorer study habits, and experience difficulty completing tests and assignments on time. Consistent with this, DuPaul et al. (2021) found that college students with ADHD received lower GPAs, engaged in fewer study skills strategies, made slower progress in their programs, and tended to persist in their programs for fewer semesters than their non-ADHD peers. Such inconsistencies and methodological issues in the literature reflect findings that a majority of studies investigating ADHD in college students continue to suffer from a number of methodological shortcomings (DuPaul et al., 2009; Green & Rabiner, 2012). Specifically, many studies of post-secondary students rely on cross-sectional designs and small convenience samples, which limit the generalizability of results as well as limiting conclusions on the developmental course of these deficits throughout post-secondary years. Moreover, a majority of studies in this area have tended to use GPA as the sole measure of academic success in post-secondary (typically over just a term or two) without considering other aspects of academic success (e.g., graduation rates, time to graduate, course withdrawals; DuPaul et al., 2021), as well as relying on self-reported GPAs which are subject to response biases. In order to overcome these methodological limitations, researchers have called for more longitudinal studies on the academic success of post-secondary students with ADHD, using multiple objective academic success indicators, as well as employing larger more representative samples (DuPaul et al., 2009, 2021; Gormley et al., 2019; Green & Rabiner, 2012).

In addition, despite inconsistent findings regarding gender differences in ADHD symptomatology, including various studies finding men may have greater, equal, and sometimes less ADHD symptomatology than women (Gomez, 2016; Williamson & Johnston, 2015; Young et al., 2020), few studies have examined the academic effects of ADHD symptomatology in men and women separately. Moreover, few studies have evaluated the relative contributions of specific dimensions of ADHD symptomatology (Schwanz et al., 2007), instead choosing to examine overall ADHD symptomatology by using total symptom scores or not controlling for shared variability in statistical analyses when specific symptom dimensions are separately examined. The potentially unique effects of ADHD symptomatology on academic outcomes for men and women, as well as the relative importance of specific dimensions as predictors of various academic success outcomes (e.g., GPA, graduation rates) clearly warrant further research attention.

## Present Study

This study used a longitudinal design to examine the relationship between ADHD symptomatology and academic success in a large sample of post-secondary undergraduate students. Given the limitations of existing research on this topic in this population, the present study had two broad objectives: (1) Examine the relationship between ADHD symptomatology and a variety of objective academic success indicators (e.g., GPA, graduation rates) in a large sample of emerging adults separately for men and women, and (2) evaluate the relative importance of different core symptom dimensions of ADHD (i.e., inattention, hyperactivity, impulsivity) as predictors of academic success indicators separately for men and women.

## Method

### Participants

Participants were 3,688 first-year undergraduate students (1,024 men and 2,664 women) from three consecutive cohorts of full-time students attending a small liberal arts university in Central Ontario. To control for the effect of age on ADHD symptomatology, only participants between the ages of 18 to 25 years (*M* = 19.29, *SD* = 1.18) at the start of their studies were included. The majority of participants (87%) were Caucasian, with 5% reporting Asian ethnicity, 2% African, 1% Hispanic, 1% Native, and 4% Other. Participants were from a diverse range of academic programs at the university, including the sciences (e.g., biology, environmental sciences), social sciences (e.g., sociology, business administration), and humanities (e.g., cultural studies, history).

### Measures

#### Conners adult ADHD rating scale (CAARS)

The Conners Adult ADHD Rating Scale (CAARS; Conners et al., 1999) is a 66-item self-report measure of adult ADHD symptomatology. Respondents are asked to respond to each item using a 4-point Likert scale, ranging from zero to three (0 = “not at all, never,” 3 = “very much, very frequently”). The CAARS consists of nine subscales which assess a variety of ADHD-related symptoms. For the present study, only data for the 9-item inattention and 9-item hyperactivity-impulsivity scales (adapted from DSM-IV criteria for ADHD) were used. Furthermore, to allow for examinations of the unique contributions of the hyperactivity and impulsivity dimensions, the nine hyperactivity-impulsivity items were also used to create two separate subscales for these symptoms (six items for hyperactivity and three items for impulsivity). Mean symptom scores for each dimension were used to reduce the effects of the non-normality of the data. High scores on each of the CAARS scales indicates a high level of ADHD symptomatology (Conners et al., 1999). Cronbach’s alphas for each of the inattention, hyperactivity-impulsivity, and total ADHD scales were .77, .74, and .83, respectively.

#### Academic success

Academic success was measured objectively using official academic records accessed through the University Registrar’s Office. Academic success variables included cumulative final grade point averages (GPAs) and degree completion status (i.e., complete vs. incomplete).

### Procedures

During undergraduate orientation week in the first year of study, participants were recruited for the present study. Participation was voluntary; however, many participants were compensated with randomly drawn prizes. For each cohort, over 95% of students provided informed consent and participated in the study, thus providing a representative sample of the population of university students at the host university. Demographic information (e.g., gender, date of birth, student identification number) was collected using a brief 8-item questionnaire. Participants then completed the 66-item CAARS as part of a larger battery of self-report measures. Six-years following this initial data collection phase, academic records were matched (using student ID numbers) to each participant’s scores on the CAARS. Six-year graduation rates are a common bench-mark for monitoring or comparing academic achievement rates in Canada and the United States (Qin & Phillips, 2019). The study was approved by the university’s Research Ethics Board (REB).

#### Statistical procedures

##### Effect of ADHD symptoms on degree completion

To assess the effects of ADHD symptoms on university graduation rates and maximize the ease of interpretation, a gender by graduation status by ADHD symptom type mixed ANOVA was conducted with mean-item scores for each of the three ADHD symptom scales as the dependent variable.

##### Effect of ADHD symptoms on final grade point average

To assess the effects of ADHD symptoms on the final GPAs of students who completed their degrees, several structural equation models were tested. In the first series of structural equation models, a single latent ADHD symptom variable, constructed from scores on the three ADHD subscales, was used to predict final GPAs separately by gender. In a second series of structural equation models, three correlated latent variables for inattention, hyperactivity, and impulsivity symptoms, constructed from items on each of the three ADHD subscales, were used to predict final GPAs, separately by gender.

Estimation of each model was done using the Asymptotic Distribution-Free Gramian (ADFG) estimation method in order to account for the ordinal nature of the indicator variables. The following goodness-of-fit indices were used to evaluate model fit: the McDonald Fit Index (MFI), the Standardized Root Mean-Squared Residual (SRMR), and the Root Mean-Square Error of Approximation (RMSEA). Given the lack of universally accepted “gold standards” for interpreting goodness-of fit indices (Kline, 2011), the following graded fit criteria were used based on previously recommended cut-offs (Browne & Cudeck, 1993; Hu & Bentler, 1999; Sivo et al., 2006): MFI ≥ 0.90, SRMR ≤ 0.08, RMSEA ≤ 0.05 for good fit; MFI ≥ 0.87, SRMR ≤ 0.10, RMSEA ≤ 0.08 for acceptable fit. Additionally, magnitudes of individual parameter estimates (e.g., expected factor loadings ≥0.30; Brown, 2006) and standardized residuals were examined to identify potential sources of misfit in the models.

## Results

### Effects of ADHD Symptomatology on University Degree Completion

Table 1 presents means and standard deviations for the ADHD scales for participants who completed their degrees and those who withdrew before completing their degrees. For the mixed ANOVA, there was a main effect for gender, *F*(1, 3684) = 19.94, *p* < .001, $\eta_{\text{p}}^{2}$ = .01, with men having higher levels of ADHD symptomatology than women. There was a main effect for the type of ADHD symptomatology, *F*(2, 7368) = 357.43, *p* < .001, $\eta_{\text{p}}^{2}$ = .09, with planned comparisons showing scores to be highest for hyperactivity symptoms, followed by inattention symptoms, and the lowest being for impulsivity symptoms. The interaction between type of ADHD symptomatology and gender was significant, *F*(2, 7368) = 39.28, *p* < .001, $\eta_{\text{p}}^{2}$ = .01, with planned comparisons showing men to have higher levels of inattention and impulsivity symptoms than women, but no gender difference for hyperactivity symptoms. There was also a significant interaction between type of ADHD symptomatology and graduation status, *F*(1, 7368) = 4.09, *p* = .017, $\eta_{\text{p}}^{2}$ = .001, with planned comparisons showing that, compared to participants who withdrew before graduating, participants who graduated had lower inattention symptoms (*d* = 0.10), but not hyperactivity or impulsivity symptoms. According to Cohen’s (1992) conventions, all significant effects were small.

**Table 1.** Means and Standard Deviations for ADHD Scales by Graduation Status and Gender.

		ADHD scale			
		ADHD	INA	HYP	IMP
	*N*	*M* (*SD*)	*M* (*SD*)	*M* (*SD*)	*M* (*SD*)
Total sample	3,688	1.02 (0.42)	1.03 (0.51)^b^	1.08 (0.50)^a^	0.83 (0.56)^c^
Men	1,024	1.07 (0.43)*	1.14 (0.52)*	1.07 (0.50)*	0.87 (0.56)*
Women	2,664	0.99 (0.42)**	0.98 (0.50)**	1.09 (0.51)*	0.81 (0.56)**
Graduated	1,621	0.99 (0.42)^†^	1.00 (0.50)^†^	1.07 (0.49)	0.82 (0.55)^†^
Men	432	1.05 (0.43)	1.11 (0.52)	1.06 (0.50)	0.88 (0.55)
Women	1,189	0.97 (0.41)	0.95 (0.48)	1.07 (0.49)	0.80 (0.54)
Withdrew	2,067	1.03 (0.43)^††^	1.05 (0.51)^†^	1.09 (0.51)	0.83 (0.57)^†^
Men	592	1.09 (0.43)	1.17 (0.51)	1.08 (0.50)	0.86 (0.57)
Women	1,475	1.01 (0.43)	1.00 (0.51)	1.10 (0.52)	0.82 (0.57)

*Note*. Superscripts denote significant mean differences with † and * indicating mean differences within columns and letters indicating mean differences within rows. ADHD = total ADHD symptomatology; INA = inattention; HYP = hyperactivity; IMP = impulsivity. Different numbers of symbols in the same series (e.g., † vs. ††, * vs. **) indicate significant mean differences.

### Effects of ADHD Symptomatology on University Graduates’ Final GPAs

**Figure 1.**
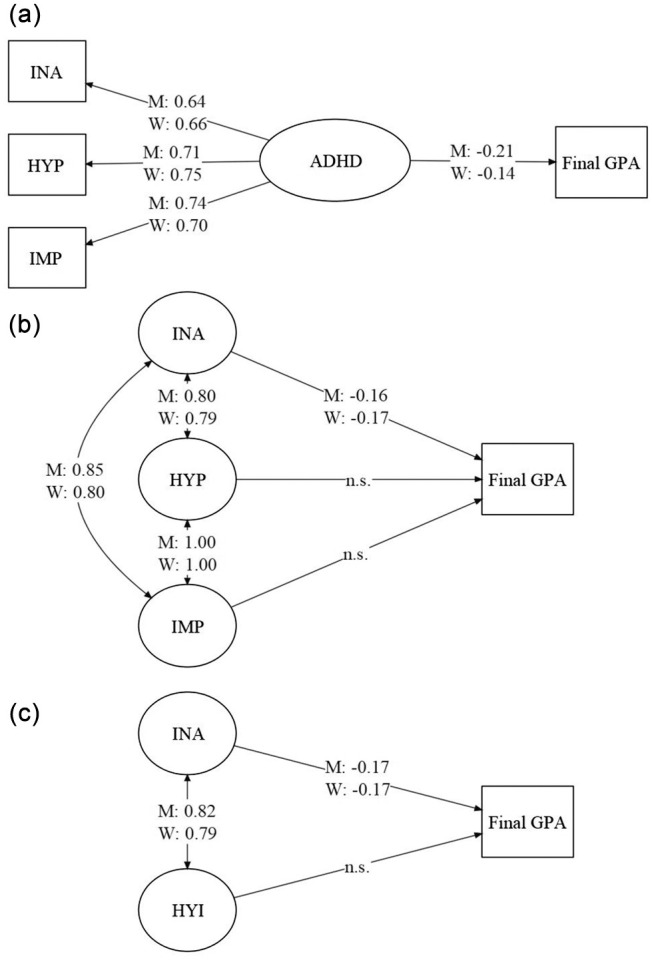
Structural equation models for the relationship between final GPA and (a) a single latent variable for ADHD, (b) three latent variables for inattention, hyperactivity, and impulsivity symptom dimensions, or (c) two latent variables for inattention and hyperactivity-impulsivity for men and women. Item factor loadings are not presented for simplicity. *Note*. All parameters presented are significant at *p* < .001 unless otherwise indicated. INA = inattention; HYP = hyperactivity; IMP = impulsivity; HYI = hyperactivity-impulsivity; M = men; W = women; n.s. = not significant.

Results for the structural equation models are presented in Figure 1. The models for total ADHD symptomatology showed overall good fit for men and women separately. For men, MFI = 0.99, SRMR = 0.04, RMSEA = 0.11, RMSEA 90% CI [0.06, 0.17], and for women, MFI = 0.99, SRMR = 0.03, RMSEA = 0.08, RMSEA 90% CI [0.05, 0.11]. As is evident from Figure 1a, results of the models indicated total ADHD symptomatology was a significant, modest predictor (parameter ranging from −0.14 to −0.21) of lower final GPAs.

The models for the three correlated ADHD symptom dimensions similarly showed overall acceptable fit for men and women separately (for all item-factor loadings for each of the structural equation models, see Supplemental Table 1). For men, MFI = 0.75, SRMR = 0.11, RMSEA = 0.06, RMSEA 90% CI [0.05, 0.07], and for women, MFI = 0.83, SRMR = 0.08, RMSEAS = 0.05, and RMSEA 90% CI [0.05, 0.06]. As is evident from Figure 1b, results of the models indicated only the inattention symptom dimension was a significant, modest predictor (parameter ranging from −0.16 to −0.17) of lower final GPAs. Both the hyperactivity and impulsivity dimensions were not significant predictors in any of the models. Moreover, as evident from Figure 1b, inter-factor parameter estimates between the hyperactivity and impulsivity dimensions, for both men and women, indicated the two dimensions were redundant with each other. Thus, a two-factor model was tested by combining items from the two dimensions to represent a single hyperactivity-impulsivity factor.

Overall, the two-factor models with two correlated ADHD symptom dimensions showed identical fit to the three-factor models, likewise indicating acceptable fit for men and women separately. For men, MFI = 0.75, SRMR = 0.11, RMSEA = 0.06, RMSEA 90% CI [0.05, 0.07], and for women, MFI = 0.83, SRMR = 0.08, RMSEA = 0.05, RMSEA 90% CI [0.05, 0.06]. Consistent with the three-factor models, as is evident from Figure 1c, results for the two-factor models indicated only the inattention symptom dimension was a significant, modest predictor (−0.17) of lower final GPAs.

## Discussion

The aim of the present study was to improve on the methodological shortcomings and identified gaps in the current literature on the relationship between ADHD symptomatology and academic success in post-secondary education. We utilized a longitudinal design and robust latent-variable data analytic strategy to examine the relative contributions of different core symptom dimensions of ADHD (i.e., inattention, hyperactivity, impulsivity) as predictors of a variety of academic success indicators in a large sample of students. In general, our findings support the view that individuals with higher levels of ADHD symptomatology at the start of their academic program show poorer long-term academic success, regardless of gender. However, there is a differential impact of specific dimensions of ADHD symptomatology.

The most consistent finding is the important role that inattention symptoms play in the academic success of both men and women, regardless of how academic success is operationalized. For instance, results regarding student degree completion showed that students who withdrew before completing their degrees had significantly greater inattention symptoms, but not different hyperactivity or impulsivity symptoms, than students who completed their degrees. Although there are many reasons for why students may choose to not complete their degrees, these findings are consistent with previous research that has demonstrated an increased likelihood of those with higher levels of ADHD symptoms to withdraw from classes and drop out of post-secondary education completely (DuPaul et al., 2009; Hechtman et al., 2016; Prevatt & Young, 2014). However, our findings highlight the primary role that inattention symptoms play in this vulnerability to withdrawing from post-secondary education and constitutes a unique finding relative to the current literature on student retention among adults with ADHD. For educators and educational institutions, this finding underscores the importance of assessing inattention problems among students at the start of their post-secondary programs. Early intervention programming (e.g., coaching and/or academic skills training) could help reduce dropout rates and increase student retention for at risk students. Particularly for students with individual education plans (IEPs) and a diagnosis of ADHD with a predominantly inattentive symptom presentation, these results stress the need for educational institutions to focus programming and academic accommodations on students’ inattention problems, which appear to be explicitly linked to overall academic success (DuPaul et al., 2017; Kim & Lee, 2016).

Beyond degree completion rates, we were also interested in examining whether ADHD symptomatology, and specific core symptom dimensions, would predict academic success for students who successfully completed their degrees. Results from a series of structural equation models showed that overall ADHD symptomatology is indeed a modest predictor of final GPA, regardless of gender. However, consistent with our findings regarding degree completion, results from both three-factor models (i.e., inattention, hyperactivity, and impulsivity) and two-factor models (i.e., inattention and hyperactivity-impulsivity) of ADHD revealed that only inattention symptoms emerged as a significant (modest) predictor of final GPAs. These findings are consistent with previous research that has pointed to ADHD symptomatology as contributing to a continued impairment in academic achievement from childhood and adolescence to adulthood (DuPaul et al., 2021; Prevatt & Young, 2014). Moreover, our findings are also consistent with previous studies that have similarly examined the relative contributions of different ADHD symptom dimensions to academic outcomes in post-secondary students (Schwanz et al., 2007). For instance, Schwanz et al. (2007) found that, when differentiating between inattention and hyperactivity-impulsivity symptom dimensions in a hierarchical multiple regression, inattention symptoms contributed the most to the prediction of first year first-semester GPAs. However, like many other studies in the current literature, Schwanz et al. utilized a much smaller sample of students who ranged in ages from 18 to 46 years old and measured academic success over only a single semester.

Despite previous research highlighting inconsistencies in the prevalence of ADHD symptomatology among men and women (Gomez, 2016; Williamson & Johnston, 2015), the consistency between our findings from models using men and women separately indicates that the observed pattern of negative impacts of ADHD symptoms, and inattention symptoms specifically, on academic success across the post-secondary experience are not specific to just men or women. At least in part, this lack of gender differences may be a consequence of our use of objective measures of academic success (i.e., official academic records of final GPAs and graduation status), rather than subjective measures (e.g., self-reported GPAs and academic concerns), as objective measures are largely found to demonstrate fewer gender differences due to ADHD (Williamson & Johnston, 2015).

### Implications for Educational Programming

Taken together, these findings underscore the important role that inattention symptoms of ADHD can play in the cumulative struggles that many students face over the course of their post-secondary experiences. Consistent with this perspective are the findings of qualitative studies that demonstrate the many ways symptoms of inattention may impact post-secondary students with ADHD on a day-to-day basis (Kwon et al., 2018; Lefler et al., 2016). For instance, Kwon et al. (2018) and Lefler et al. (2016) have both consistently found that students with ADHD report difficulties with inattention-related academic problems, including organization, planning, and time management skills, as well as procrastination, sustained attention, and distractibility. Each of these academic-related problems would increase the struggle and length of time it may take students with ADHD to complete their coursework and their degrees, if they complete them at all. Indeed, a study by Adler et al. (2017) found inattention symptoms to have considerable overlap with various executive function (EF) difficulties (e.g., time mismanagement, trouble planning ahead, multitasking) which have previously been shown to predict academic underachievement in post-secondary students with or without an ADHD diagnosis (Biederman et al., 2006). In the context of educational institutions, these results suggest that psychoeducational programing might want to specifically target inattention symptoms in those at-risk for academic underachievement, regardless of prior ADHD diagnosis (e.g., test and assignments accommodations and coaching; DuPaul et al., 2017; Kim & Lee, 2016).

A number of recent evaluations of educational programs specifically designed to enhance students’ organizational skills have been shown to be effective interventions for students with ADHD (Fabiano & Pyle, 2019). For instance, a recent study conducted by Bettis et al. (2017) found preliminary evidence for the effectiveness of EF training in reducing students’ ADHD symptoms as well as symptoms of anxiety and various executive function difficulties (e.g., behavioral inhibition, emotional control, planning, and organization). Similarly, LaCount et al. (2018) found that an organizational skills training intervention was effective in reducing college students’ ADHD symptoms, as well as improving their use of EF skills, including organizational, time management, and planning skills. Given the effectiveness of these educational programs, and others such as peer mentoring (Fox et al., 2010), in reducing ADHD symptoms and improving inattention-related problems among college students with ADHD, the results of the present study overall suggest the potential utility of implementing these programs for at risk students. Although it should be noted that more research is needed to determine whether these reductions in inattention symptoms following educational programs ultimately lead to greater academic success for college students with ADHD.

### Strengths, Limitations, and Future Directions

Despite the important implications of the present study, our results should be understood in the context of a number of limitations. Firstly, the present study made use of self-reported ADHD symptoms, which may be subject to response bias. For instance, Millenet et al. (2018) found that adults tended to report fewer ADHD symptoms and less impairment relative to their parent’s ratings. Thus, future studies may benefit from using both self-reports and observer reports of ADHD symptomatology in order to reduce bias in reporting. Another limitation of the present study is our use of a non-clinical sample. Although the use of a non-clinical sample when examining post-secondary samples is common in the general ADHD literature, university and college students with diagnosed ADHD have higher levels of ADHD symptomatology than those in the general population of post-secondary students. Thus, future research would benefit from using clinical samples of university students diagnosed with ADHD and longitudinal designs to improve generalizability of their results to clinical populations.

Additionally, analyses in the present study did not control for a number of other variables known to effect academic success in university students, including symptoms of anxiety and depression, learning disorders, substance and medication use, and IQ (DuPaul et al., 2021). Future studies should attempt to replicate our results using models controlling for these other known contributors to academic success outcomes. It should also be noted that our measure of ADHD symptomatology, the CAARS, was developed based on DSM-IV criteria for ADHD (Conners et al., 1999) and therefore does not take advantage of enhanced wording in the DSM-5 which is thought to better capture ADHD symptoms as presented by adults (Lefler et al., 2020). This limitation may partly explain our findings regarding the lack of an association between the hyperactivity-impulsivity dimension and academic success indicators. However, previous research with the DSM-5 criteria for ADHD has consistently found inattention symptoms to be more central and predictive of ADHD diagnosis and functional impairment in adults than hyperactivity-impulsivity (Matte et al., 2015).

Nonetheless, the results of the present study add to the literature on adult ADHD symptomatology in a number of ways. Firstly, this study made several improvements on the methodological shortcomings of studies in the current literature, including using a large, representative sample of first year undergraduate students, a longitudinal design, and a robust assessment of academic success indicators (e.g., multiple indicators and official academic records), as well as restricting analyses to a homogeneous sample of emerging adults (i.e., ages ranging from 18 to 25 years). These methodological improvements on previous studies allowed for more generalizable conclusions about post-secondary students in general and a more robust examination of the relationships between ADHD symptoms and academic success across the post-secondary experience. The present study also adds to the existing literature by demonstrating the important role that inattention symptoms play as the primary driver of the relationship between ADHD symptomatology and academic success in post-secondary education settings, regardless of gender, suggesting it as a priority focus for intervention programming.

## Supplemental Material

sj-docx-1-jad-10.1177_10870547211036758 – Supplemental material for ADHD and Academic Success in University Students: The Important Role of Impaired Attention Click here for additional data file. Supplemental material, sj-docx-1-jad-10.1177_10870547211036758 for ADHD and Academic Success in University Students: The Important Role of Impaired Attention by Colin Henning, Laura J. Summerfeldt and James D. A. Parker in Journal of Attention Disorders
