# Supplementary material for: ADHD and Academic Success in University Students: The Important Role of Impaired Attention
Source: J Atten Disord. 2021 Aug 12;26(6):893–901. doi: 10.1177/10870547211036758 (PMC8859654; doi:10.1177/10870547211036758)
Supplement: sj-docx-1-jad-10.1177_10870547211036758 – Supplemental material for ADHD and Academic Success in University Students: The Important Role of Impaired Attention [file sj-docx-1-jad-10.1177_10870547211036758.docx]

**Supplementary Materials**

| Supplementary Table 1. | | | | | | | | | | | | | |
| --- | --- | --- | --- | --- | --- | --- | --- | --- | --- | --- | --- | --- | --- |
| *Standardized Parameter Estimates from the Two-Factor and Three-Factor ADHD Structural Equation Models for Men and Women* | | | | | | | | | | | | | |
| CAARS Item | Three-Factor Models | | | | | | |  | Two-Factor Models | | | | |
|  | Men | | |  | Women | | |  | Men | |  | Women | |
|  | INA | HYP | IMP |  | INA | HYP | IMP |  | INA | HYI |  | INA | HYI |
| 1 | 0.63 |  |  |  | 0.53 |  |  |  | 0.64 |  |  | 0.53 |  |
| 24 | 0.69 |  |  |  | 0.66 |  |  |  | 0.69 |  |  | 0.66 |  |
| 29 | 0.70 |  |  |  | 0.58 |  |  |  | 0.70 |  |  | 0.58 |  |
| 33 | 0.53 |  |  |  | 0.52 |  |  |  | 0.53 |  |  | 0.52 |  |
| 42 | 0.62 |  |  |  | 0.60 |  |  |  | 0.63 |  |  | 0.60 |  |
| 48 | 0.43 |  |  |  | 0.48 |  |  |  | 0.44 |  |  | 0.48 |  |
| 60 | 0.68 |  |  |  | 0.66 |  |  |  | 0.69 |  |  | 0.66 |  |
| 64 | 0.65 |  |  |  | 0.65 |  |  |  | 0.65 |  |  | 0.65 |  |
| 65 | 0.75 |  |  |  | 0.69 |  |  |  | 0.76 |  |  | 0.69 |  |
| 9 |  | 0.36 |  |  |  | 0.44 |  |  |  | 0.36 |  |  | 0.44 |
| 14 |  | 0.42 |  |  |  | 0.57 |  |  |  | 0.42 |  |  | 0.57 |
| 21 |  | 0.54 |  |  |  | 0.58 |  |  |  | 0.53 |  |  | 0.58 |
| 38 |  | 0.39 |  |  |  | 0.29 |  |  |  | 0.38 |  |  | 0.29 |
| 41 |  | 0.64 |  |  |  | 0.59 |  |  |  | 0.64 |  |  | 0.58 |
| 50 |  | 0.75 |  |  |  | 0.73 |  |  |  | 0.75 |  |  | 0.73 |
| 22 |  |  | 0.48 |  |  |  | 0.50 |  |  | 0.49 |  |  | 0.51 |
| 58 |  |  | 0.52 |  |  |  | 0.45 |  |  | 0.53 |  |  | 0.46 |
| 62 |  |  | 0.65 |  |  |  | 0.54 |  |  | 0.66 |  |  | 0.54 |
| *Note.* All presented parameter estimates were statistically significant (p < .001). INA = Inattention; HYP = Hyperactivity; IMP = Impulsivity; HYI = Hyperactivity-Impulsivity | | | | | | | | | | | | | |
